# StressDB: A Locally Installable Web-Based Relational Microarray Database Designed for Small User Communities

**DOI:** 10.1002/cfg.153

**Published:** 2002-04

**Authors:** Madhusmita Mitra, Nigam Shah, Lukas Mueller, Scuth Pin, Nina Fedoroff

**Affiliations:** 1 519 Wartik Laboratory The Pennsylvania State University University Park Pennsylvania 16802 USA; 2 Carnegie Institution Department of Plant Biology 260 Panama Street Stanford CA 94305 USA

## Abstract

We have built a microarray database, StressDB, for management of microarray data from our studies on stress-modulated genes in Arabidopsis. StressDB provides small user groups with a locally installable web-based relational microarray database. It has a simple and intuitive architecture and has been designed for cDNA microarray technology users. StressDB uses Windows^™^ 2000 as the centralized database server with Oracle^™^ 8i as the relational database management system. It allows users to manage microarray data and data-related biological information over the Internet using a web browser. The source-code is currently available on request from the authors and will soon be made freely available for downloading from our website athttp://arastressdb.cac.psu.edu.


**Comparative and Functional Genomics**
*Comp Funct Genom* 2002; **3**: 91–96.
Published online 11 March 2002 in Wiley InterScience (www.interscience.wiley.com). **DOI:** 10.1002/cfg.153

**Research Article**

### StressDB: A locally installable web-based relational microarray database designed for small user communities

Madhusmita Mitra[1], Nigam Shah[1], Lukas Mueller[2], Scuth Pin[1] and Nina Fedoroff[1]*
[1] *519 Wartik Laboratory, The Pennsylvania State University, University Park, Pennsylvania 16802, USA*
[2] *Carnegie Institution, Department of Plant Biology, 260 Panama Street, Stanford, CA 94305, USA*

*Correspondence to:
519 Wartik Laboratory, The Pennsylvania State University, University Park, Pennsylvania 16802, USA.
E-mail: mxm66@psu.edu*

Received: 31 October 2001
Accepted: 12 February 2002

### Abstract

**We have built a microarray database, StressDB, for management of microarray data from our studies on stress-modulated genes in Arabidopsis. StressDB provides small user groups with a locally installable web-based relational microarray database. It has a simple and intuitive architecture and has been designed for cDNA microarray technology users. StressDB uses Windows™ 2000 as the centralized database server with Oracle™ 8i as the relational database management system. It allows users to manage microarray data and data-related biological information over the Internet using a web browser. The source-code is currently available on request from the authors and will soon be made freely available for downloading from our website athttp://arastressdb.cac.psu.edu. Copyright © 2002 John Wiley & Sons, Ltd.**

**Keywords:** **microarray database; gene expression; *Arabidopsis*; plant stress**

### Introduction

DNA microarray technology is rapidly increasing in popularity for measuring the transcript levels of many genes simultaneously. The large amounts of data generated by microarray experiments have created new demands for data management and analysis tools. Flat-files and spreadsheets are no longer capable of handling the volume of data that is being generated or making the detailed queries users require, necessitating the use of relational database management systems. On the basis of the size and specificity of the audience they cater to, microarray databases can be divided into three categories. Arranged in the decreasing order of audience size and increasing order of audience specificity are (i) those available for public data submission (Gene Expression Omnibus at the National Center for Biotechnology Information (NCBI):**http://www.ncbi.nlm.nih.gov/geo/**; Array Express at the European Bioinformatics Institute (EBI):**http://www.ebi.ac.uk/arrayexpress/**), (ii) those that have been implemented within an institution and are available for public query (Stanford Microarray Database (SMD) at Stanford University:**http://www.dnachip.org/**; Sherlock *et al.*, 2001; ChipDB at the Whitehead Institute at MIT:**http://young39.wi.mit.edu/chipdb_public/**; Gene Expression Database (GXD) at the Jackson Laboratory:**http://www.informatics.jax.org/**; RNA Abundance Database (RAD) at University of Pennsylvania:**http://www.cbil.upenn.edu/RAD2/**) and (iii) those that have been designed for local installation by individual groups (Another Microarray Database (AMAD) at Stanford/Berkeley/UCSF:**http://www.microarrays.org/index.html** and GeneX at NCGR: **http://genex.ncgr.org/**; Mangalam *et al.*, 2001).

Public microarray repositories, like Array Express at EBI and GEO at NCBI, are designed with the highest degree of flexibility for microarray gene-expression data storage from different organisms subjected to a variety of treatments. Since the microarray data may be collected using different microarray technologies, the database needs to be structured to accommodate raw and processed data in a way that is independent of the underlying technology. Microarray databases implemented on an institutional or laboratory scale are specialized

Copyright © 2002 John Wiley & Sons, Ltd.

to meet the needs of a specific user community. Institutional microarray databases more or less closely resemble the public repositories depending upon the breadth of the database users, determined by the variety of organisms under study, the microarray technologies used, and data analysis and query features used by the investigators. For example, although SMD, which is currently the largest institutional database, resembles public repositories in its capability to store microarray data from many users and multiple organisms, it is microarray technology-specific and handles only 2-channel glass slide microarray data.

The database community may be sub-divided into groups of specialized users with similar needs in terms of organisms of interest, gene-related information content, microarray experiment-related information content, data query and analysis features and the use of a single microarray technology. Locally installable databases designed for such user groups differ from public and institutional databases in two ways. First, they have a simpler, albeit more specialized backend architecture, which allows for a simpler application-programming interface (API). The result is a simpler database for a specific user community that is both easier to manage and easier to use. Second, their backend structure permits more detailed queries. Because the user group is small, these databases can include tables with more specific data columns/fields to permit more detailed queries better tailored to the needs of the users. Storage of microarray data from a large user community within a single database is made possible at the cost of abstraction of the information content available for query. However, data abstraction comes at the cost of a lower query resolution. For example, the details on how reference and treated samples are generated, including the type of tissue, the developmental stage, the treatment, and the quality of the RNA sample, are likely be stored in a single field (such as 'sample_description') in a public or institutional database, rather than in separate fields. This structure allows the storage of details on the generation of samples from any source and any sample treatment. Because the level of resolution of a database query is limited to the resolution of the fields of the table, storing sample information in single field permits comparing the 'sample_description' field across experiments, but precludes stringent queries based upon information contained within the field. If the information on sample generation were stored in multiple fields, it would be possible to search across experiments based on those fields. Data abstraction would permit the storage of experimental details in a manner that is independent of the 'details' of the information, which is an important consideration for public and institutional databases but not for databases that cater to specialized user groups.

The locally installable microarray databases that exist currently include AMAD, GeneX, maxdSQL (**http://bioinf.man.ac.uk/microarray/maxd/maxdSQL/**), GeneDirector (**http://www.biodiscovery.com/**) and GeNet (**http://www.csa.ru/Inst/gorb_dep/inbios/genet/genet.htm**). MaxdSQL and GeneX have been designed for local installation on a public or an institutional scale. MaxdSQL is the ANSI SQL 92 compliant, relational database management system (rdbms)-independent SQL script for a database backend, based on the Array Express database schema. The Array Express schema is designed for a public repository. Its microarray technology-independent schema and emphasis on data abstraction for housing information from varied sources and treatments are similar to those of a well-designed public repository. Hence maxdSQL is not well suited for small user groups. The architecture of GeneX resembles that of Array Express and is similarly not well suited for a small user community. AMAD is a platform independent flat-file database for analyzing microarray data that seamlessly integrates with data analysis software such as Cluster and Treeview. While it is more appropriate for a small user group, AMAD's flat-file architecture limits its data querying capabilities and precludes associating biological information with microarray data. The commercial microarray databases available for local installation, GeneDirector and GeNet, are proprietary and their internal structure is not available for public scrutiny.

Because local databases are meant to meet the needs of a specific microarray user group, it is unlikely that a single microarray database architecture will meet the needs of all user groups because the very size and complexity of such an architecture will be a liability for any single user group. Thus the availability of a variety of specialized, yet easily customized local microarray databases, is likely to best serve the needs of the research community. As various flavors of microarray databases become freely available, making one's own will involve taking the closest template database already freely available, one that meets personal user needs and

Copyright © 2002 John Wiley & Sons, Ltd. 

resource limitations, and customizing it to meet specific needs.

To meet the data management needs of our lab, we built StressDB (**http://arastressdb.cac.psu.edu/**), a relational database that allows us to store cDNA microarray data and related biological information on plant stress-modulated genes in *Arabidopsis thaliana*. StressDB has a simple relational schema that is better suited for our specialized user group than the schemas of the existing locally installable databases. The relational schema can support detailed queries across experiments based upon specific experimental conditions, sample conditions, spot/gene-related information and experimental data. Despite the fact that the Windows platform is the most widely used among biologists, the microarray database community has largely ignored it from a centralized server standpoint. While databases have been designed for XWindows-based platforms like Linux (GeneX) and SQL scripts for the database schema for maxdSQL are portable across RDBMSs that can run on Windows® platforms, most web-based microarray databases are implemented on Unix servers – servers that are significantly less used by biologists. Although the knowledge of operating system administration is imperative for maintaining a database server, Windows offers a more familiar learning environment for most biologists. StressDB runs on a Windows® 2000 workstation and allows users to load, retrieve and analyze microarray data via the Internet using a web browser. The database backend structure itself is organism-independent and permits the storage of cDNA microarray data and gene-related information from any organism. With minor modifications of the fields in some of the tables( e.g., replacing the 'soil_conditions' field in our RNA_sample information table with 'growth_media' for yeast data), our database can be customized to manage cDNA microarray data and related information from any organism. In order to make biological data comparison across technologies possible, StressDB also stores gene expression information as ratios of experimental and reference samples from other sources e.g. data generated using cDNA microarray technology by other groups. Although the gene expression ratios from data generated by oligonucleotide arrays may be stored, a lot of the information for these types of microarrays (for example: the absolute intensity values which are meaningful for oligonucleotide arrays) is not recorded. StressDB complies with the current MIAME v1.0 standards (**http://www.mged.org/Annotations-wg**).

### Methods

StressDB uses Oracle® 8i as its database management system (**http://www.oracle.com/**). We are using a Dell XPS T700r machine with Windows® NT workstation (**http://www.microsoft.com**) as our operating system and are running Apache 1.3.12 (**http://apache.org/**) as our web server. Scripts are written in Perl (**http://www.perl.com/**). In addition we are using the database interface (DBI) (**http://search.cpan.org/search?module = DBI**), the common gateway interface (CGI) (**http://stein.cshl.org/WWW/software/CGI/**) and the graphical display (GD) (**http://stein.cshl.org/WWW/software/GD/**) modules to make all the connectivities. We have obtained a license for using Xcluster and related programs from the original authors (Sherlock *et al.*, 2001; **http://genome-www.stanford.edu/~sherlock/cluster.html**).

### Database architecture and implementation

### Database backend

The backend structure is relational and has been designed for the storage of biological information about the cDNAs spotted on glass slides, details on the experiments including probe generation and hybridization, microarray images and both raw and user-normalized experimental data. Information about each experiment is stored in four database tables: the experiment_set, experiment, experiment_data and rna_sample tables ( Figure 1). At the top of the hierarchy is the experiment_set table, each entity in which is linked to one or more entities in the experiment table. The experiment_set table contains the common information of a group of experiments that logically belong together, for example a group of experiments that are part of a time series. The table contains information on the hybridization conditions of the experiments and links to the print job and author tables. A single row in the experiment table, an experiment, is linked to as many rows in the experiment_data table as the spots on the microarray chip used to perform the experiment. Thus, the smallest unit of experimental information, a single spot on a chip, can be tracked for any given experiment stored in

Copyright © 2002 John Wiley & Sons, Ltd.

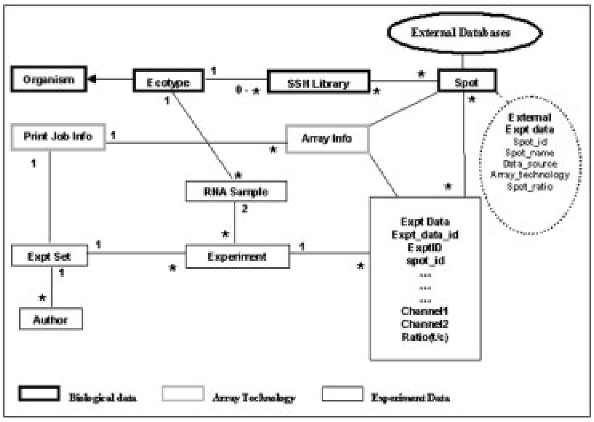

**Figure 1.** The data model. This schematic shows the various types of information stored in StressDB and the connectivities among them. Each information bin translates into a database table. Each table contains various types of information, stored in the columns of the table (not shown, detailed schema will be made available at **http://stressdb.biotec.psu.edu/**). Each row within a database table describes completely an entity stored within the database table. Two database tables are linked via a common column. The relationship between two tables can be described with the numbers adjacent to the line joining them. For example, the relationship between the 'Ecotype'table and the 'subtractive-hybridization or SSH library' table reads as 'each row in the SSH Library table is linked to one and only one row in the Ecotype table whereas each row in the Ecotype table can be linked to zero or more rows in the SSH Library table'

the database. Each entity in an experiment table is also linked to 2 entities in the rna_sample table corresponding to the two channels. The experiment table also contains a description column and a link to the overlay image for the experiment. The rna_sample table contains all the details that are recorded by the experimenters about treatment conditions of the specimen from which RNA was isolated. The rna_sample, experiment_set and experiment tables contain all the information about a single experiment stored in a manner that allows queries involving specific experimental conditions. The experiment information is linked to the array information such as the microarray print job and detailed array layout information. Each of the entities in the experiment data table, the spots on the chip, is also linked to the relevant biological information (Figure 1) on the spot table. A detailed schema showing all the attributes of the tables can be found at our website (**http://arastressdb.cac.psu.edu**).

### Application programming interface (API)

The application-programming layer (middleware) enables the backend to communicate with the front end. It has been written in Perl and communicates with the database using the DBI module and with the browser using the CGI and GD modules. User authentication, web-based or direct loading of data by the users into the database, data normalization and processing of the front-end queries from users are all part of the middleware and have been written in Perl with embeddedSQL database queries.

### Query interface

We are using an Apache web server. Since most of the scripts are generated dynamically or on the fly, the front end is tightly integrated with the middleware and has been written in Perl and dynamic HTML. The front end allows privileged users to load their microarray data over the web. The database has private and public domains visible to the database users and the public, respectively. Depending upon viewer privileges, a different set of experiments will be displayed to the browser. Data will be made accessible to the public at the discretion of the user or upon publication. Detailed query pages allow users to retrieve specific information from one or more experiments, cluster genes and experiments, re-normalize raw data using one of the available normalization methods or a user defined normalization factor and perform some statistical analyses on the data. Figure 2 shows a user's personal page displaying all the experiments loaded by the user into the database in a tabular form. This page gets dynamically updated as soon as the user loads data into the database. Each row of the table describes an experiment briefly. Each row contains links to detailed information about the experimental conditions, RNA samples, raw and processed gene-expression data and microarray printing. This page also takes the user to the data analysis forms for gene clustering.

### Data analysis

### Data loading

Registered users can upload data, related experimental information and images directly into the database over the Internet using a web browser.

Copyright © 2002 John Wiley & Sons, Ltd. 

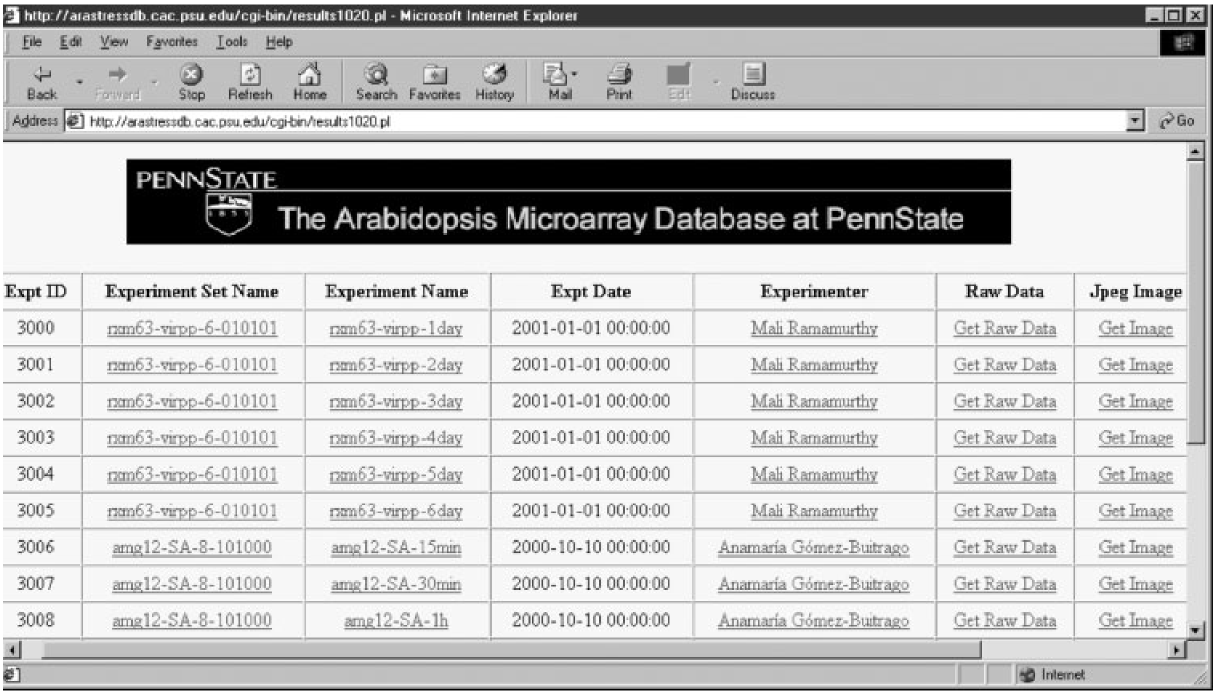

| Expt ID | Experiment Set Name | Experiment Name | Expt Date | Experimenter | Raw Data | Jpeg Image |
|---|---|---|---|---|---|---|
| 3000 | rxm63-virpp-6-010101 | rxm63-virpp-1day | 2001-01-01 00:00:00 | Mali Ramamurthy | Get Raw Data | Get Image |
| 3001 | rxm63-virpp-6-010101 | rxm63-virpp-2day | 2001-01-01 00:00:00 | Mali Ramamurthy | Get Raw Data | Get Image |
| 3002 | rxm63-virpp-6-010101 | rxm63-virpp-3day | 2001-01-01 00:00:00 | Mali Ramamurthy | Get Raw Data | Get Image |
| 3003 | rxm63-virpp-6-010101 | rxm63-virpp-4day | 2001-01-01 00:00:00 | Mali Ramamurthy | Get Raw Data | Get Image |
| 3004 | rxm63-virpp-6-010101 | rxm63-virpp-5day | 2001-01-01 00:00:00 | Mali Ramamurthy | Get Raw Data | Get Image |
| 3005 | rxm63-virpp-6-010101 | rxm63-virpp-6day | 2001-01-01 00:00:00 | Mali Ramamurthy | Get Raw Data | Get Image |
| 3006 | amg12-SA-8-101000 | amg12-SA-15min | 2000-10-10 00:00:00 | Anamaría Gómez-Buitrago | Get Raw Data | Get Image |
| 3007 | amg12-SA-8-101000 | amg12-SA-30min | 2000-10-10 00:00:00 | Anamaría Gómez-Buitrago | Get Raw Data | Get Image |
| 3008 | amg12-SA-8-101000 | amg12-SA-1h | 2000-10-10 00:00:00 | Anamaría Gómez-Buitrago | Get Raw Data | Get Image |

**Figure 2.** A user's experiment folder. This is a screenshot of a dynamically generated experiment page that is displayed after a registered user logs into the database and chooses to view experiments. The page gets dynamically updated as soon as data gets loaded into the database. Data is displayed in a tabular form, each row describing an experiment performed by the user. The rows contain hyperlinks to additional details on the experiments or to data analysis (**http://arastressdb.cac.psu.edu**)

Data normalization is performed at the time of data loading and both the raw and normalized data are stored in the database. The three most common normalization methods, spiking control, housekeeping genes and whole genome methods (Hegde *et al.*, 2000) have been incorporated into the database and can be selected at the time of data upload. Users can also specify their own normalization factors.

### Gene clustering

Cluster analysis is commonly used to reveal similarities among gene expression profiles. Among the commonly used clustering algorithms are hierarchical clustering (Eisen *et al.*, 1998), k-means (Tavazoie *et al.*, 1999) singular value decomposition or SVD (Holter *et al.*, 2000) and self-organizing maps or SOMs ( Tamayo, *et al.*, 1999). StressDB uses Xcluster and related programs (Sherlock *et al.*, 2001) to allow both genes and experiments to be clustered using hierarchical clustering and SOMs. Images are dynamically generated and displayed to the browser using the GD module (Figure 3).

### Clone search

Our cDNA microarrays comprise of *Arabidopsis thaliana* ESTs. We retrieve information about these clones from public databases (TAIR: **http://www.arabidopsis.org/**, Huala *et al.*, 2001 and GenBank: **http://www.ncbi.nlm.nih.gov/Genbank**). The information retrieved is parsed and stored in the Spot table (Figure 1). StressDB permits clone information to be retrieved by clone name, stress library name and clone sequence. We have installed NCBI's blast server (**http://www.ncbi.nlm.nih.gov/BLAST/**) and allow users to carry out BLAST and MEGA-BLAST searches against our clones. The clones are linked to external databases that can provide more information about them.

### Future directions

We are packaging our database scripts for installation by other labs (upon the receipt of appropriate licenses for the required software from the respective companies). Scripts will be open source and

Copyright © 2002 John Wiley & Sons, Ltd. 

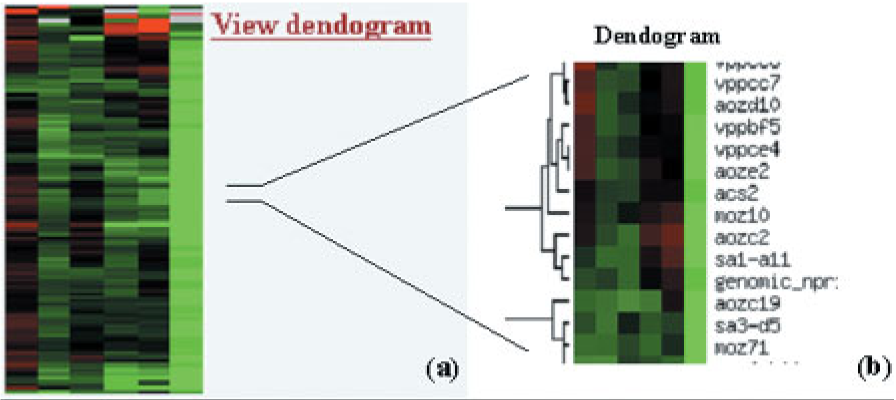

**Figure 3.** Screen shot of data analysis. This screen shot shows a time-course experiment performed by a registered user (a) that has been subjected to hierarchical clustering using Xcluster (Sherlock *et al.*, 2001) and displayed to the browser using the GD module. The 'view dendogram'; link takes us to the dendogram (b), which shows us a detailed image of the clustered genes including information about the genes that was requested by the user for display available for downloading from our website. Xcluster needs to be licensed from the original authors. Currently, the gene information in our database is limited to information retrieved from external databases that gets parsed and stored in our database. Associating information from literature on the genes represented on the microarrays with the gene expression data is an important and challenging problem. Besides being able to efficiently manage microarray data, we are working towards making more effective connections between expression data of the spotted genes and the biological information known about them. We are committed to meeting the evolving community standards. StressDB is compliant with MIAME v 1.0 standards. NetGenics (**http://www.netgenics.com/software.html**), the microarray gene expression database group (MGED) at the EBI (**http://www.ebi.ac.uk/microarray/MGED/**), and Rosetta Inpharmatics (**http://www.rii.com/**) are among the key players in the development of standards for the microarray community and are currently jointly working towards such standards. In the coming years we have been assured of stable data annotation tools which will make it easier to implement these standards. StressDB will continue to evolve to comply with community standards and be compatible with public repositories for easy data transfer after publication.

### References

AMAD at Stanford/Berkeley/UCSF: **http://www.microarrays.org/index.html**.

Array Express at EBI **http://www.ebi.ac.uk/arrayexpress/**.

ChipDB at the Whitehead Institute at MIT: **http://young39.wi.mit.edu/chipdb_public/**.

Eisen MB, Spellman PT, Brown PO, Botstein D. 1998. Cluster analysis and display of genome-wide expression patterns. *Proc Natl Acad Sci U S A* **95**: 14863–14868.

Gene Expression Omnibus at NCBI: **http://www.ncbi.nlm.nih.gov/geo/**.

GeneDirector: **http://www.biodiscovery.com/**.

GeNet: **http://www.csa.ru/Inst/gorb_dep/inbios/genet/genet.htm**.

GeneX at NCGR: **http://genex.ncgr.org/**.

GXD at the Jackson Laboratory: **http://www.informatics.jax.org/**.

Hegde P, Qi R, Abernathy K, Gay C, *et al.* 2000. A concise guide to cDNA microarray analysis. *Biotechniques* **29**: 548–550, 552–554, 556.

Holter NS, Mitra M, Maritan A, *et al.* 2000. Fundamental patterns underlying gene expression profiles: simplicity from complexity. *Proc Natl Acad Sci U S A* **97**: 8409–8414.

Huala E, Dickerman A, Garcia-Hernandez M, *et al.* 2001. The *Arabidopsis* Information Resource (TAIR): A comprehensive database and web-based information retrireview, analysis, and visualization system for a model plant. *NucleicAcids Res* **29**: 102–105.

Mangalam HJ, Stewart J, Zhou J, *et al.* 2001. GeneX: An Open Source gene expression database and integrated tool set. *IBM systems journal* **40**: 552–569.

MaxdSQL: **http://bioinf.man.ac.uk/microarray/maxd/maxdSQL/**.

MIAME: **http://www.mged.org/Annotations-wg/**.

RAD at University of Pennsylvania: **http://www.cbil.upenn.edu/RAD2/**.

Sherlock G, Hernandez-Boussard T, Kasarskis A, *et al.* 2001. The Stanford Microarray Database. *Nucleic Acids Res* **29**: 152–155.

SMD at Stanford University: **http://www.dnachip.org/**.

Tamayo P, Slonim D, Mesirov J, Zhu Q, *et al.* 1999.Interpreting patterns of gene expression with self-organizing maps: methods and application to hematopoietic differentiation. *Proc Natl Acad Sci U S A* **96**: 2907–2912.

Tavazoie S, Hughes JD, Campbell MJ, *et al.* 1999. Systematic determination of genetic network, architecture. *Nature Genetics* **22**: 281–285.

XCluster: **http://genome-www.stanford.edu/~sherlock/cluster.html**.

Copyright © 2002 John Wiley & Sons, Ltd. 

## References

[PDF_00463] Eisen M. B., Spellman P. T., Brown P. O., Botstein D. (1998). Cluster analysis and display of genome-wide expression patterns.. Proc Natl Acad Sci U S A.

[PDF_00471] Hegde P., Qi R., Abernathy K., Gay C., Dharap S., Gaspard R., Hughes J. E., Snesrud E., Lee N., Quackenbush J. (2000). A concise guide to cDNA microarray analysis.. Biotechniques.

[PDF_00474] Holter N. S., Mitra M., Maritan A., Cieplak M., Banavar J. R., Fedoroff N. V. (2000). Fundamental patterns underlying gene expression profiles: simplicity from complexity.. Proc Natl Acad Sci U S A.

[PDF_00477] Huala E., Dickerman A. W., Garcia-Hernandez M., Weems D., Reiser L., LaFond F., Hanley D., Kiphart D., Zhuang M., Huang W. (2001). The Arabidopsis Information Resource (TAIR): a comprehensive database and web-based information retrieval, analysis, and visualization system for a model plant.. Nucleic Acids Res.

[PDF_00487] Sherlock G., Hernandez-Boussard T., Kasarskis A., Binkley G., Matese J. C., Dwight S. S., Kaloper M., Weng S., Jin H., Ball C. A. (2001). The Stanford Microarray Database.. Nucleic Acids Res.

[PDF_00491] Tamayo P., Slonim D., Mesirov J., Zhu Q., Kitareewan S., Dmitrovsky E., Lander E. S., Golub T. R. (1999). Interpreting patterns of gene expression with self-organizing maps: methods and application to hematopoietic differentiation.. Proc Natl Acad Sci U S A.

[PDF_00495] Tavazoie S., Hughes J. D., Campbell M. J., Cho R. J., Church G. M. (1999). Systematic determination of genetic network architecture.. Nat Genet.

